# Supplementary material for: Digital finance and performance in innovation: Evidence from universities
Source: PLoS One. 2023 Sep 20;18(9):e0289958. doi: 10.1371/journal.pone.0289958 (PMC10511105; doi:10.1371/journal.pone.0289958)
Supplement: S1 File — (PDF) [file pone.0289958.s001.pdf]

editage

Editing Certificate

This document certifies that the manuscript listed below has been edited to ensure language and grammar accuracy and is error free in these aspects. The edit was performed by professional editors at Editage, a division of Cactus Communications. The author's core research ideas were not altered in any way during the editing process. The quality of the edit has been guaranteed, with the assumption that our suggested changes have been accepted and the text has not been further altered without the knowledge of our editors.

MANUSCRIPT TITLE

Digital Finance and Performance in Innovation and Entrepreneurship: Evidence From Higher Education

AUTHORS

Jialu You

ISSUED ON

April 20, 2023

JOB CODE

PQRHU_5

THE EDITAGE Promise QUALITY GUARANTEE

Vikas Narang
Chief Operating Officer - Editage

editage

Editage, a brand of Cactus Communications, offers professional English language editing and publication support services to authors engaged in over 1300 areas of research. Through its community of experienced editors, which includes doctors, engineers, published scientists, and researchers with peer review experience, Editage has successfully helped authors get published in internationally reputed journals. Authors who work with Editage are guaranteed excellent language quality and timely delivery.

GLOBAL :
+1(833) 979-0061 | request@editage.com

CHINA :
400-120-3020或 021-6020-9400 | fabiao@editage.cn

CACTUS

impact.science

researcher.life

lifesciences.cactusglobal.com

editage.com | editage.co.kr | editage.in | editage.cn | editage.com.br | editage.com.tw | editage.de
