# Supplementary material for: Digital finance and performance in innovation: Evidence from universities
Source: PLoS One. 2023 Sep 20;18(9):e0289958. doi: 10.1371/journal.pone.0289958 (PMC10511105; doi:10.1371/journal.pone.0289958)
Supplement: Attachment — Submitted filename: Respond lists.docx [file pone.0289958.s002.docx]

**a Response to Reviewers letter**

Dear Editors and Reviewers:

Thank you for your letter and the reviewers' comments concerning our manuscript entitled " Digital Finance and Performance in Innovation: Evidence from Universities" (Manuscript ID:2231410). Those comments are valuable and helpful for improving our paper and the essential guiding significance to our research. We have substantially revised manuscripts comments by the reviews. Modified portions are marked in red in the article. The primary corrections in the form and the responses to the reviewer's comments as follow: Answer to the reviewer's 1 comments: Review comments

1. Reviewer #1: Thank you for revising the paper. However, I still have concerns:1) I don't find H1, the paper now begins with H2 page 13.

Answer: thanks reviewer suggestion! I am so sorry about this mistake caused by polish article. We have already added the mission hypothesis 1 as following and rewritten sector 2:

## 2.1 Digital finance and innovation performance

Innovation is one of the driving factors of economic and social development, and the Austrian economist Schumpeter's "innovation theory" defines innovation as a new combination of production factors. It is an effective way to cultivate innovative talents (Śledzik, Karol,2013). The training of innovative talents is inseparable from financial support, and there is an important economic relationship between digital finance and the performance of innovation and entrepreneurship in colleges and universities.

In view of the uneven and insufficient development of traditional financial system, the long fund flow cycle of universities and the insufficient supply of funds to universities in less developed areas restrict the improvement of innovation and entrepreneurship performance (Hamburg, I,2017). The vigorous development of information technology has given birth to digital finance as an emerging financial service model. Digital inclusive finance has a positive impact on financial inclusion and stability, provides a basis for innovation, and can effectively increase the investment in innovation and entrepreneurship in universities. Combing the existing studies (Dykes, E,2013; Stefaniak, J,2019).

The impact of digital inclusive finance on the performance of university innovation and entrepreneurship can be summarized as follows: digital finance can accurately assess the default risk of customers through big data modeling, and carry out real-time and dynamic supervision of credit customers, effectively control and identify risks, avoid adverse selection and moral risks in the financial market, improve risk control ability, explore more business space, and bring more new opportunities for university innovation and entrepreneurship .It brings more new opportunities for university innovation and entrepreneurship (Becker,2018). At the same time, digital finance also effectively reduces the cost of information search and matching between borrowers and lenders. Goldfarb & Tucker (2019) pointed out that big data can effectively reduce economic costs and help improve the performance of university innovation and entrepreneurship. Finally, the development of digital finance provides a good financial environment for university innovation and entrepreneurship. Trinugroho, I (2020) suggest that Digital inclusive finance has bred a distributed business pattern with strong inclusiveness and permeability, which is conducive to the communication and cooperation of innovation subjects in the distributed innovation network, constructing a collaborative innovation mechanism and helping to improve the quality of university innovation and entrepreneurship.

In addition, the combination of traditional finance such as payment, investment and credit with digital technology effectively improves the convenience of customers using financial services and provides a good external environment for financial services to improve the performance of university innovation and entrepreneurship. In summary, this study proposed the following research hypothesis 1.

*H1: Digital finance can effectively promote the improvement of innovation and entrepreneurship.*

**2. the innovation and entrepreneurship performance index used is questionable. The authors use variables which aren't reflecting the actual innovation and entrepreneurship related output. output indicators refer to talent development, scientific research and social services which is common practice. but which of these indicators is considered to explain the 1) innovation performance and 2) entrepreneurship performance? both indicators (or if you like the combined indicator) don't reflect innovation and entrepreneurship outcomes at all.**

Answer: thanks reviewer suggestion! Regarding the selection of indicators is based on the literature for the selection of indicators. This paper adopts the reviewer's opinion that the selected indicators in this paper cannot respond to the entrepreneurial performance, first, change the innovation and entrepreneurship performance of universities into innovation performance; second, add the literature basis and support for the selection of innovation performance indicators, and rewrite the analysis and literature theory of innovation performance indicators selection still as following:

The index data related to colleges and universities used in this study were mainly obtained from the 2010–2019 Compilation of Basic Statistics on Colleges and Universities of the Ministry of Education, and the city-level data were obtained from the China City Statistical Yearbook for the year’s corresponding to the period of interest. The final sample comprised 72 colleges and universities’ 10-year panel data.

The independent variable was tertiary institutions’ innovation. The extant literature suggests the convenience of using human capital, financial investment, and research investment indicators to closely examine higher education innovation capabilities (María Leticia 2020). In this study, based on the existing literature (Yang Chen 2015), we divide the university inputs into three major inputs of human input, financial input, and material input.

Based on Xiong (2019), we measure university innovation performance outputs into three major outputs of talent development, scientific research, and Innovation education effects. In terms of human input, considering that colleges and universities are the main places for innovation knowledge production and innovation talent cultivation, the main work of their workers can be divided into innovation teaching and research, administration, and auxiliary support.

So according to the different types of work, this paper selects three indicators for the innovation human input of colleges and universities: the number of full-time teachers, the number of administrative staff, and the number of teaching and auxiliary staff. In terms of financial input, innovation research outcomes in universities are closely linked to financial investment (Fu, Y. 2023). This paper refers to the general division method of income sources of colleges and universities and divides the financial input of colleges and universities into three items: state financial allocation, education business income of colleges and universities and other income. Among them, the state financial appropriation includes education business appropriation, scientific innovation research fund appropriation, other appropriation, etc.; the education business income of colleges and universities mainly refers to the tuition fee income of colleges and universities; other income refers to other non-financial and non-education business income of colleges and universities, such as innovation business income, contribution from affiliated units and social donation.

Material input. Innovation education effects is an important indicator of innovation performance (Vincent-Lancrin, S. 2017). In his work propose a model of innovation performance where the probability of being innovative performance depends on the innovation education effects. They examine whether the technology transfer, patent and consultancy adopted can explain different types of innovation performance at universities. Dutta et al (2011) analyze the relationship between innovation material input and innovation education effects. Therefore, the innovation material input of colleges and universities mainly includes three aspects: one is the input of fixed assets such as innovation teaching buildings, libraries, student dormitories and canteens; the second is the input of library literature; the third is the input of instruments and equipment necessary for teaching and research. Accordingly, this paper selects 3 indicators of fixed assets formation, books and teaching and research instruments and equipment value to portray the physical investment of universities. The output indicators include talent cultivation, scientific research, and social service.

**3.based especially on 2 I don't consider the econometric analysis being useful and vaild. the variables used in my view don't respond to the intended innovation and entrepreneurship performance. in other words, the research framework isn't solid.**

**Answer:** To address the problem that the empirical analysis does not reflect entrepreneurial performance, this paper accepts the opinion of the reviewers and changes the title of the paper to "Digital Finance and Performance in Innovation: Evidence from Higher Education ". The selection of innovation performance indicators is supported by the literature. Based on Xiong (2019), we measure university innovation performance outputs into three major outputs of talent development, scientific research, and Innovation education effects. In terms of human input, considering that colleges and universities are the main places for innovation knowledge production and innovation talent cultivation, the main work of their workers can be divided into innovation teaching and research, administration, and auxiliary support.

So according to the different types of work, this paper selects three indicators for the innovation human input of colleges and universities: the number of full-time teachers, the number of administrative staff, and the number of teaching and auxiliary staff. In terms of financial input, innovation research outcomes in universities are closely linked to financial investment (Fu, Y. 2023). This paper refers to the general division method of income sources of colleges and universities and divides the financial input of colleges and universities into three items: state financial allocation, education business income of colleges and universities and other income. Among them, the state financial appropriation includes education business appropriation, scientific innovation research fund appropriation, other appropriation, etc.; the education business income of colleges and universities mainly refers to the tuition fee income of colleges and universities; other income refers to other non-financial and non-education business income of colleges and universities, such as innovation business income, contribution from affiliated units and social donation.

Material input. Innovation education effects is an important indicator of innovation performance (Vincent-Lancrin, S. 2017). In his work propose a model of innovation performance where the probability of being innovative performance depends on the innovation education effects. They examine whether the technology transfer, patent and consultancy adopted can explain different types of innovation performance at universities. Dutta et al (2011) analyze the relationship between innovation material input and innovation education effects. Therefore, the innovation material input of colleges and universities mainly includes three aspects: one is the input of fixed assets such as innovation teaching buildings, libraries, student dormitories and canteens; the second is the input of library literature; the third is the input of instruments and equipment necessary for teaching and research. Accordingly, this paper selects 3 indicators of fixed assets formation, books and teaching and research instruments and equipment value to portray the physical investment of universities. The output indicators include talent cultivation, scientific research, and social service.

Reference:

[1] Vincent-Lancrin, S., G. Jacotin, J. Urgel, S. Kar and C. González-Sancho (2017), Measuring Innovation in Education: A Journey to the Future, OECD Publishing, Paris.

[2] Dutta, D., Li, J., and Merenda, M. (2011). Fostering entrepreneurship: impact of specialization and diversity in education. International Entrepreneurship and Management Journal, 7(2):163–179.

[3] Fu, Y. (2023). The impact of government funding on research innovation: An empirical analysis of Chinese universities. Managerial and Decision Economics, 44(1). 285– 296.

[4] Xiong (2019). The construction of innovation and entrepreneurship teaching performance evaluation index system in colleges and universities and its application. Journal of Innovation and Enterprise Education.10(1): 674-893.
